# Mitochondrial DNA Deletions and Plasma GDF-15 Protein Levels Are Linked to Hormonal Dysregulation and Multi-Organ Involvement in Female Reproductive Endocrine Disorders

**DOI:** 10.3390/life15111744

**Published:** 2025-11-13

**Authors:** Vera Varhegyi, Barnabas Banfi, Domonkos Trager, Dora Gerszi, Eszter Maria Horvath, Miklos Sipos, Nandor Acs, Maria Judit Molnar, Szabolcs Varbiro, Aniko Gal

**Affiliations:** 1 Institute of Genomic Medicine and Rare Disorders, Semmelweis University, 1085 Budapest, Hungary; varhegyi.vera@semmelweis.hu (V.V.); banfi.barnabas@phd.semmelweis.hu (B.B.);; 2 Department of Obstetrics and Gynecology, Semmelweis University, 1085 Budapest, Hungary; 3 Department of Physiology, Semmelweis University, 1085 Budapest, Hungary; 4 HUN-REN Multiomics Neurodegeneration Research Group, Hungarian Research Network, 1085 Budapest, Hungary; 5 Department of Obstetrics and Gynecology, University of Szeged, 6725 Szeged, Hungary; 6 Workgroup for Science Management, Doctoral School, Semmelweis University, 1085 Budapest, Hungary

**Keywords:** PCOS, POI, insulin resistance, GDF-15, mitochondrial dysfunction, mtDNA deletion

## Abstract

Mitochondrial dysfunction contributes to female reproductive endocrine disorders and is frequently associated with multisystem symptoms. Insulin resistance (IR) is a common metabolic disorder strongly linked to polycystic ovary syndrome (PCOS), while premature ovarian insufficiency (POI) also impairs fertility. Mitochondrial DNA (mtDNA) deletions and the stress-responsive cytokine growth differentiation factor 15 (GDF-15) have recently emerged as complementary biomarkers of mitochondrial impairment. In this retrospective observational study, we examined reproductive hormones, plasma GDF-15, mtDNA deletions, and clinical symptoms in insulin-resistant women, including those with PCOS or POI. Eighty-one patients were divided into three subgroups: IR-only (*n* = 49), IR-PCOS (*n* = 19), and IR-POI (*n* = 13). IR was defined based on elevated insulin levels during oral glucose tolerance testing (>10 mU/L at 0 min, >50 mU/L at 60 min, >30 mU/L at 120 min) according to national gynecological endocrinology guidelines, acknowledging that IR is not universally accepted as a distinct clinical entity. POI was defined as reduced ovarian reserve before age 40 with anti-Müllerian hormone (AMH) <1.0 ng/mL. Clinical symptoms were assessed using a questionnaire, medical record, and physical examination. MtDNA deletions were detected by long-range PCR, and GDF-15 was measured by ELISA. Free thyroxine (T4) emerged as an independent predictor of GDF-15, suggesting that thyroid function modulates mitochondrial stress signaling in insulin-resistant women. MtDNA deletions and/or elevated GDF-15 correlated with endocrine, gastrointestinal, and neuropsychiatric symptoms, and reduced AMH/FSH ratios indicated impaired ovarian function. Cross-sectional analysis further revealed lower AMH and AMH/FSH ratios in older women with mtDNA deletions, consistent with a trend toward accelerated reproductive aging. Overall, these findings support the role of GDF-15 and mtDNA deletions as complementary biomarkers of mitonuclear stress, with potential relevance for both systemic and reproductive health.

## 1. Introduction

Metabolic and endocrine factors substantially contribute to infertility, with insulin resistance (IR) and polycystic ovary syndrome (PCOS) representing prevalent and treatable conditions [1]. Insulin resistance (IR) is a prevalent metabolic state and an important risk factor for common endocrine and metabolic disorders. Reported prevalence estimates vary depending on the population and diagnostic criteria, ranging from 15.5% to 46.5% [2,3]. Although IR is not a universal, consensus-based, uniform diagnostic entity, it is widely used in clinical and research settings, particularly in reproductive endocrinology [4,5]. In women of reproductive age, IR is defined as impaired responsiveness of peripheral tissues—such as muscle, adipose tissue, and liver—to insulin, leading to compensatory hyperinsulinemia. IR is often associated with obesity and PCOS and has been linked to impaired ovulatory function, reduced fertility, and an increased risk of long-term complications such as type 2 diabetes mellitus (T2DM), metabolic syndrome, and cardiovascular disease, all of which have a substantial global impact [6,7,8]. This dysfunction is commonly associated with conditions like polycystic ovary syndrome (PCOS) and may impair ovulatory function, fertility, and increase the risk of metabolic and cardiovascular disorders. A diagnosis of PCOS is made according to the 2003 Rotterdam criteria, which require the presence of two or more of the following three characteristics: oligomenorrhea, hyperandrogenism (either clinical or biochemical), and an ultrasonographic image of polycystic ovaries. It is often accompanied by obesity and mild inflammation. Polycystic ovary syndrome (PCOS) is a prevalent condition affecting 5 to 15% of women within their reproductive age. [9]. There is a strong correlation between the two diseases, as insulin resistance is highly prevalent among women with PCOS [7,10].

It is also possible that mitochondrial dysfunction plays a role in the pathogenesis of IR-PCOS. The ATP level and mitochondrial membrane potential were found to be significantly reduced in cells derived from patients with IR-PCOS, while increased reactive oxygen species (ROS) levels were observed in cells from the IR-PCOS group [11]. Moreover, insulin resistance is associated with reduced mitochondrial plasticity, which ultimately results in diminished insulin-stimulated mitochondrial activity. This can result in an increase in lipid metabolites, such as acyl coenzyme A (acyl-CoA), diacylglycerol (DAG), and ceramides, which can further contribute to the development of insulin resistance [12]. The capacity for mitochondrial plasticity, including metabolic flexibility, represents the limiting factor for in vivo ATP synthesis rates in insulin-resistant humans [13,14].

Premature ovarian insufficiency (POI) is a clinical condition characterized by the loss of normal ovarian function in women under the age of 40. This can lead to infertility and hormonal imbalances. The etiology and pathogenesis of POI are multifactorial, including genetic, autoimmune, iatrogenic, and environmental factors [15]. Several factors, including genetic predisposition, autoimmune responses, environmental exposures, and medical interventions, influence the complex etiology and pathogenesis of IR, PCOS, and POI. A comprehensive understanding of these factors is essential for developing targeted therapies and improved diagnostic approaches for affected women [16,17]. Oxidative stress, which frequently contributes to mitochondrial dysfunction, is also regarded as a principal factor in ovarian aging. It is hypothesized that this is caused by the potential accumulation of ROS, which leads to mitochondrial dysfunction and, thus, to cellular apoptosis, especially in oocytes that are highly sensitive to oxidative damage [18,19,20].

GDF-15 is currently the most widely reported and diagnostically robust plasma biomarker of mitochondrial dysfunction, with consistent evidence across adult and pediatric populations [21,22]. Recently, the plasma levels of GDF-15 and the presence or absence of mtDNA deletions were analyzed for the first time as potential biomarkers of mitochondrial dysfunction underlying insulin resistance, polycystic ovary syndrome, and associated infertility. Patients in the study group exhibited significantly elevated plasma GDF-15 levels and higher rates of mtDNA deletions compared to healthy controls [3]. In addition to its diagnostic function, GDF-15 is a stress-responsive cytokine belonging to the TGF-β superfamily. It integrates mitochondrial, metabolic, and immune stress signals, thereby linking mitochondrial dysfunction to systemic endocrine and inflammatory pathways [21,22,23]. To clarify our usage of terms, it is essential to note that when referring to the mtDNA deletion itself, we use the term “mtDNA deletion.” Alternatively, when evaluating the process, we referred to mitochondrial dysfunction.

This study aimed to characterize the distribution of mitochondrial dysfunction (as assessed by mtDNA deletions) and related multi-organ involvement in insulin-resistant women, whether or not they had PCOS or POI. We also examined the association between female reproductive hormones and plasma GDF-15, as well as the association between these measures and the presence of mtDNA deletions. The primary objective was to evaluate associations within the entire cohort. Subgroup comparisons, stratified by mtDNA deletion status, were pre-specified as hypothesis-generating to explore potential biological heterogeneity.

## 2. Materials and Methods

### 2.1. Studied Cohort

The study population consisted of female outpatients examined at the Department of Obstetrics and Gynecology at Semmelweis University for infertility, insulin resistance, and associated polycystic ovary syndrome or premature ovarian insufficiency. The patients were presenting with symptoms or complaints affecting multiple organ systems concurrently. The patients were selected from the Polycystic Ovary Syndrome, Mitochondrial Dysfunction, Obesity, Insulin Resistance, Infertility (POMODORI) Cohort (ClinicalTrials.gov Identifier: NCT06167135) and the NEPSYBANK of the Institute of Genomic Medicine and Rare Disorders, Semmelweis University. Patients were enrolled in the study between 2022 and 2024, and blood and urine samples were collected from them at the time of enrollment for genetic and biochemical testing. The inclusion criteria were as follows: women of reproductive age (20–45 years) presenting with insulin resistance (IR), with or without a confirmed diagnosis of PCOS or POI, who provided written informed consent for molecular genetics and biochemical testing. Exclusion criteria comprised pregnancy, an active infection, malignancy or an acute inflammatory disease at the time of sampling. Women who had received hormonal or metabolic therapy within three months prior to enrolment were also excluded. All patients provided written informed consent prior to sampling and molecular genetic testing. The patients participated in the present investigation voluntarily. The clinical status of patients and healthy control volunteers was evaluated by administering a comprehensive clinical questionnaire and a physical examination. The study was approved by the Hungarian National Centre for Public Health (15672-6/2022/ECIG). All trial participants, including the control group, were provided with pre-test clinical genetic counseling. To achieve the scientific objectives of the study, a molecular genetic study was conducted on each patient. Patients found to be pregnant at the time of sampling were excluded from the study, as plasma GDF-15 levels are known to be elevated during pregnancy.

In our study, we defined insulin resistance as a carbohydrate metabolism disorder in which serum insulin levels measured during an oral glucose tolerance test were above 10 mU/L at 0 min, above 50 mU/L at 60 min, and above 30 mU/L at 120 min in female patients of reproductive age, in accordance with the recently published guidelines of the Hungarian Society for Obstetric and Gynecological Endocrinology [24]. The diagnosis of PCOS was established based on the 2003 Rotterdam criteria. The term POI was used to refer to a condition in which a woman under 40 years of age had a reduced ovarian reserve, as indicated by an anti-Müllerian hormone (AMH) value below 1.0 ng/mL.

Of the 81 individuals included in the study, 49 exhibited insulin resistance (IR) as a standalone condition, 19 individuals concurrently presented with both IR and polycystic ovarian syndrome (PCOS), and 13 patients had IR and premature ovarian insufficiency (POI) at the same time. The patients in the study ranged in age from 20 to 45 years, with a mean age of 35.38 ± 6 (CI 95: 34.23–36.53) years.

Patients were administered a preliminary clinical questionnaire (Supplementary Documents S1 and S2) as part of the initial phase of the study. The questionnaire inquired about the initial symptoms that prompted the clinical investigation, as well as whether any additional symptoms affecting various organ systems might be present. As part of the preliminary screening questionnaire, participants were required to indicate whether they had experienced any of the listed symptoms, which could potentially indicate the presence of underlying medical conditions. These included cardiovascular involvement, digestive issues, visual and auditory impairment, muscular complaints, exercise intolerance, early childhood developmental delays in speech or movement, and a history of transient ischemic attack (TIA) or stroke, ataxia, peripheral neuropathy, etc. The occurrence of psychiatric symptoms, including instances of memory impairment, the persistence of mood disorders, and possible psychotic episodes, was considered. The potential involvement of autoimmune disorders (Hashimoto-thyreoiditis, systemic lupus erythematosus, vitiligo, ankylosing spondylitis, antiphospholipid syndrome, and non-differential autoimmune syndrome) was mentioned in the questionnaires and medical documentation. Also, other endocrine abnormalities beyond those initially identified in the research objectives (hypothyroidism, hyperprolactinemia, pituitary adenoma, or hypercortisolism) were evaluated. Lastly, high intolerance to either heat or cold was also considered a potential contributing factor.

The study encompassed the gathering of routine laboratory data from female patients. The laboratory data collected included serum thyroid hormones (TSH, T4, T3), vitamin D3 levels, AMH, Follicle-stimulating hormone (FSH), Luteinizing Hormone (LH), estradiol, progesterone, and total testosterone levels. In terms of female sex hormone levels, AMH, FSH, LH, estradiol, and prolactin levels were measured between days 2 and 4 of the menstrual cycle, and progesterone levels were measured between days 22 and 24 of the menstrual cycle. Additionally, the results of oral glucose tolerance tests (fasting, 60, and 120 min serum glucose and insulin levels) and the HOMA index were recorded.

### 2.2. Molecular Genetic Analysis

#### 2.2.1. Sample Collection and DNA Analysis

Patients’ DNA and plasma samples were obtained concurrently for sample collection. For each participant, both peripheral blood and urine epithelial cell samples were collected at the same sampling time and analyzed in parallel for mtDNA deletions. Particular attention was paid to storing the plasma samples at −80 °C within one hour of sampling.

DNA was isolated from blood and urine epithelial cells [3]. Urine epithelial cells may offer a viable alternative to muscle biopsy for the detection of single mtDNA deletions, showing approximately 80% sensitivity and a significant correlation with skeletal muscle heteroplasmy levels (r ≈ 0.71, *p* ≈ 0.03) [25]. Consequently, both tissues were employed for this purpose in our patients and control subjects. The DNeasy^®^ Blood & Tissue Kit (QIAGEN GmbH, Hilden, Germany) was used to isolate DNA, following the instructions provided by the manufacturer. Briefly, DNA was lysed with Proteinase K and purified on silica spin columns according to the manufacturer’s protocol. DNA was eluted in 100 µL Buffer AE and quantified spectrophotometrically (A260/A280 ratio 1.8–2.0). Urine cell pellets were processed in parallel using the same procedure.

Isolation was performed from 100 mL of fresh urine for the analysis of urine samples. The samples were initially subjected to centrifugation at 1000× *g* for 10 min. Thereafter, the resulting pellet was washed with PBS and subjected to a second centrifugation at 1000× *g* for a further 10 min. The DNA was then isolated from the resulting cell pellet using a tissue DNA isolation kit (QIAGEN GmbH, Hilden, Germany). The DNA concentration was determined by measuring the absorbance at 260 nm using a UV spectrophotometer. The degree of purity was determined by calculating the ratio of the absorbance values at 260 nm and 280 nm.

#### 2.2.2. Analysis of mtDNA Deletion

Mitochondrial DNA (mtDNA) deletion analysis was performed in parallel on both sample types of every patient. Long-range PCR was employed to detect the 4977 bp ‘common’ deletion along with other mtDNA deletions. The amplification reactions were carried out using 1 unit of Phusion DNA polymerase (Thermo Fisher Scientific Inc., Waltham, MA, USA) in the presence of a GC-rich buffer (Phusion GC Reaction Buffer, Thermo Fisher Scientific Inc., Waltham, MA, USA), 200 µM dNTPs, and 200 pM each of the forward (5′-TAAAAATCTTTGAAATAGGGC-3′) and reverse (5′-CGGATACAGTTTTCACTTTAGCT-3′) primers. Each reaction included approximately 30 ng of DNA template and nuclease-free water, adjusted according to the DNA concentration, to a final volume of 20 µL [3]. Briefly, the PCR process was conducted using Phusion DNA polymerase under optimal conditions, with primers designed to target specific regions of the mtDNA deletion. The PCR products were then separated on a 1% high-resolution agarose gel, with a 1 kb molecular weight marker used to indicate the size of the fragments. Estimates of band sizes were used to assess fragment lengths corresponding to wild-type and deleted mtDNA. The heteroplasmy ratio, representing the proportion of wild-type to mutant mtDNA, was then quantified using QuantityOne (BIORAD, Bio-Rad Imaging Systems, Hercules, CA, USA) and ImageJ software (Quantity One^®^ 1-D Analysis Software (4.6.3 version) (Bio-Rad Imaging Systems, Hercules, CA, USA) ImageJ 1.54i software), following the established protocol [3]. In this study, the term “mtDNA deletion(s)” refers to structural damage detected by long-range PCR, while “mitochondrial dysfunction” refers to functional impairment of mitochondrial processes. Multiple mtDNA deletions are widely accepted as evidence of mitochondrial dysfunction in mtDNA maintenance disorders and may be associated with respiratory chain abnormalities. Therefore, mtDNA deletions are referred to as surrogate markers of mitochondrial dysfunction when discussing their functional consequences.

#### 2.2.3. Measurement of GDF-15 Plasma Level

Plasma GDF-15 concentrations were measured using the Human GDF-15 ELISA Kit (Thermo Fisher Scientific, BMS2258, Thermo Fisher Scientific Inc., Waltham, MA, USA) according to the manufacturer’s instructions. All samples were analyzed in duplicate, and a 7-parameter logistic calibration curve was used for quantification. Age- and sex-specific cut-off values were applied according to a meta-analysis of approximately 20,000 individuals [3,26]. To establish the GDF-15 values, a reference range was used as a point of comparison, namely a meta-analysis published in 2022 that involved approximately 20,000 individuals. This analysis determined the cut-off value for GDF-15 by age group and sex [3,26].

### 2.3. Statistical Analysis

Data are presented as the means ± S.E.M. Comparisons between two or more groups were performed using the Mann–Whitney U test and the Kruskal–Wallis Dunn’s multiple comparison test, respectively. Correlation analyses were conducted in Python (Python 3.10.5, Python Software Foundation, Wilmington, DE, USA) using the Pearsonr and Spearmanr functions, respectively, from the SciPy library (SciPy 1.9.3, SciPy Developers, NumFOCUS Inc, Austin, TX, USA). Correlations were assessed primarily using Pearson’s correlation coefficient. In addition, Spearman’s rank correlation was examined to account for the possibility of monotonic but non-linear associations. Since Spearman’s results were broadly consistent and did not alter interpretation, only Pearson’s coefficients are reported in the results. Pearson correlations were visualized with linear regression plots. To explore associations between GDF-15 and the examined parameters, both simple and multiple regression analyses were applied, adjusting for age and BMI as known confounders. Differences in proportions were assessed using the chi-square test. A *p*-value of <0.05 was considered statistically significant. Hormone levels were analyzed in patient subgroups using statistical analyses with one-way ANOVA followed by post hoc testing; *p* < 0.05, ns = not significant. Normality was assessed using the Shapiro–Wilk test prior to applying ANOVA. As this was a retrospective observational study, no formal sample size calculation was performed. Instead, all eligible patients enrolled during the study period were included to maximize the representativeness of the cohort. Primary analyses were conducted in the total cohort. Subgroup analyses stratified by mtDNA deletion status were pre-specified as exploratory owing to limited sample sizes, and corresponding findings are interpreted as hypothesis-generating.

## 3. Results

### 3.1. Investigation of Symptoms in Different Organ Systems Associated with Mitochondrial Dysfunction and Clinical Features of Patients with Elevated Plasma GDF-15 Levels

During the enrolment process, each patient completed a self-administered clinical symptom questionnaire to assess multisystem involvement (Supplementary Documents S1 and S2). Across the entire cohort, 0–7 organ systems were affected. Patients with mtDNA deletions tended to have a higher number of affected organ systems compared to deletion-negative individuals (0–7, with 13 patients having more than 5 vs. 0–5; only 1 patient had more than 5). This difference was statistically significant (χ^2^ = 6.94, *p* = 0.01). In patients with higher and elevated GDF-15 levels, organ system involvement did not differ (Supplementary Table S1).

Patients with mtDNA deletions more frequently exhibited muscle symptoms, gastrointestinal disturbances, early childhood psychomotor developmental delay, psychiatric disorders (primarily depression), autoimmune diseases (Hashimoto-thyreoiditis, systemic lupus erythematosus, vitiligo, ankylosing spondylitis, antiphospholipid syndrome, and non-differential autoimmune syndrome were mentioned in the questionnaires and medical documentation), and other endocrine abnormalities. Among these, only the prevalence of other endocrine symptoms reached statistical significance based on the chi-square test (χ^2^ = 6.45, *p* < 0.05) (Table 1). When patients were grouped by plasma GDF-15 levels, the frequency of organ-specific symptoms did not differ significantly between those with elevated and normal levels. Autoimmune involvement was numerically higher in the elevated GDF-15 group, but this did not reach significance (Table 1).

Using age-specific cutoff values for GDF-15 based on a recent meta-analysis [26] (<30 years: 2195 pg/mL; 30–39 years: 1950 pg/mL; 40–49 years: 1804 pg/mL), 12 out of 81 patients were found to have elevated GDF-15 levels. Notably, 11 of these 12 patients had an elevated BMI, and the remaining patient had a BMI at the upper limit of the normal range (25 kg/m^2^). Eight of these twelve patients with elevated GDF-15 levels also had confirmed mtDNA deletions, in line with our previous findings [3], suggesting a potential association, although the small sample size precludes statistical inference. Of the 69 patients with normal GDF-15 levels, 45 patients (65.2%) had elevated BMI (>25 kg/m^2^).

Table 2 details the symptom distribution among GDF-15–elevated cases. Among patients with elevated GDF-15, symptom profiles based on the questionnaire revealed the following: five patients experienced exercise intolerance, one had visual impairment, and five suffered from gastrointestinal complaints (Table 2). Cardiovascular symptoms were noted in only one patient, while another showed psychomotor developmental delay in early childhood. Psychiatric symptoms and thyroid or other endocrine disorders were each present in three patients, and autoimmune disorders were observed in four (33.3%). 8 of the 12 patients (66.6%) demonstrated marked intolerance to cold or heat. Importantly, none had a history of hearing loss, transient ischemic attacks/stroke, or other neurological symptoms (Table 2).

### 3.2. Distribution of Clinical Symptoms in Different Clinical Subgroups

**Figure 1:**
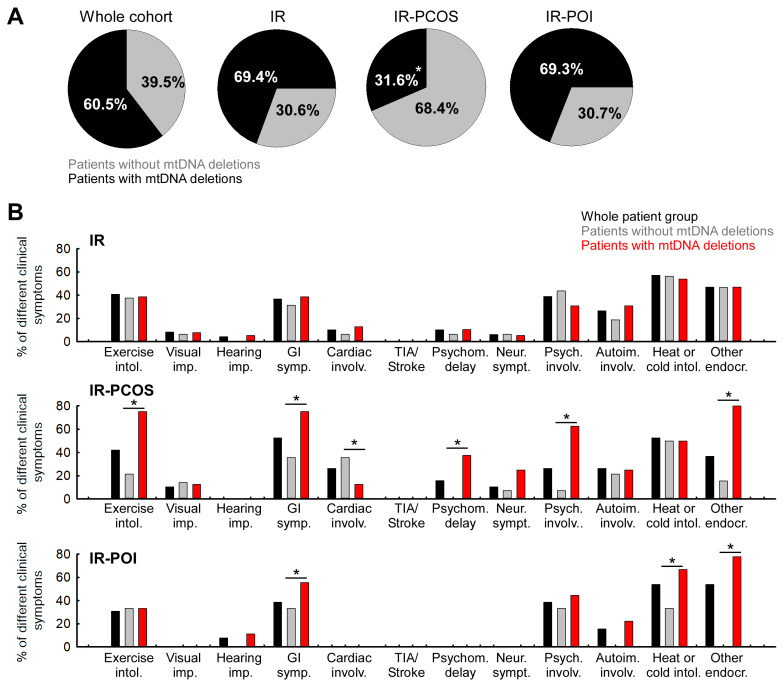
Distribution of mtDNA deletions (single/multiple) in the whole patient group and in each patient subgroup, and percentage prevalence of each organ system symptom in the IR-only, IR-PCOS, and IR-POI subgroups. (**A**) Distribution of individuals with and without single or multiple deletions of mtDNA in the whole patient group (1); in the subgroup with IR only (*n* = 49; *n*_del. neg_ = 15, *n*_del. pos_ = 34) (2); in the subgroup with both IR and PCOS (*n* = 19; *n*_del. neg_ = 13, *n*_del. pos_ = 6) (3); in the subgroup with both IR and POI (*n* = 13; *n*_del. neg_ = 4, *n*_del. pos_ = 9). The significance between pairs of groups was calculated using the chi-square test. (*: *p* < 0.05). (**B**) Prevalence of organ system symptoms by mtDNA deletion status of patients, if the group or subgroup is considered to be 100%. Percentage of organ system involvements in the whole patient group (black columns), in patients without mtDNA deletions (grey columns), and in patients with mtDNA deletions (red columns). The Mann–Whitney U test was used to determine the significance between the groups (*: *p* < 0.05). (Abbreviation: Exercise intol.: exercise intolerance; Visual imp.: visual impairment; Hearing imp.: hearing impairment; GI symp.: gastrointestinal symptoms; Cardiac involv.: cardiovascular involvement; Psychom. delay: early childhood psychomotor developmental delay; Neur. sympt.: neurological symptoms; Psych. involv.: psychiatric involvement; Autoim. involv.: autoimmune involvement; Heat or cold intol.: heat or cold intolerance; Other endocr.: other endocrine symptoms).

As previously reported [3], analysis of mtDNA deletion patterns within the present patient subgroups showed that the overall deletion rate in the current cohort was 60.5% (49 confirmed cases: 41 with multiple deletions and 8 with a single deletion). This frequency was significantly higher than the 9.7% rate observed in an age-matched healthy control group described in our earlier study [3] (Chi^2^ = 29.1, *p* < 0.05) (Figure 1A). For clarity, the control data are not displayed in Figure 1, as they derive from our previously published dataset. The deletion rate in the IR-only subgroup was 69.3%. The deletion prevalence differed markedly between the PCOS and POI subgroups, with significantly increased occurrence in the IR-PCOS group compared to the IR-POI group (31.6% vs. 69.3%, respectively) (Figure 1).

Mitochondrial dysfunction typically affects multiple organ systems and is associated with a wide range of clinical symptoms; therefore, we investigated the distribution of clinical manifestations across the different clinical subgroups. There was no difference between subgroups in the number of affected organ systems.

The symptom pattern in each subgroup revealed that visual impairment, cardiovascular involvement, and neurological problems, typically associated with aging, were most prevalent in the IR-PCOS group. In contrast, these symptoms were absent in the IR-POI patients (Table 3 and Figure 1B).

We next examined the organ-specific symptom prevalences in the IR-only, IR-PCOS, and IR-POI subgroups and their association with mtDNA deletion. Percentages were calculated relative to the number of individuals in each subgroup. Physical exercise intolerance occurred with similar frequency in the IR-only and IR-PCOS groups (40.8% and 42.1%, respectively) but was less frequent in the IR-POI group (30.8%) (Table 3). Notably, 75% of IR-PCOS patients with mtDNA deletions exhibited this symptom, while mtDNA deletion status had no influence on symptom occurrence in the IR-only and IR-POI subgroups (Figure 1B).

The overall prevalence of visual impairment was 8.2% in the IR-only group and 10.5% in the IR-PCOS group; no cases were observed in the IR-POI group (Table 3). No association was found between visual impairment and mtDNA deletion status (Figure 1B). Gastrointestinal problems were more common in the IR-PCOS group, with the highest frequency observed in those with mtDNA deletions (Table 3 and Figure 1B). Cerebrovascular symptoms, including transient ischemic attack (TIA) and stroke, were absent in all patient groups (Table 3 and Figure 1B).

Cardiovascular symptoms, delayed psychomotor development in early childhood, and neurological symptoms were absent in the IR-POI group but occurred most frequently in the IR-PCOS group (Table 3 and Figure 1B). A strong correlation was found between delayed psychomotor development, neurological involvement, and the presence of mtDNA deletions in the IR-PCOS group (Figure 1B). Psychiatric symptoms were mainly observed in the IR-only and IR-POI groups, and in these subgroups, mtDNA deletion status did not influence their presence (Figure 1B). Despite the low prevalence of psychiatric symptoms in the IR-PCOS group, a significant association with mtDNA deletions was detected (Figure 1B).

Autoimmune involvement was least prevalent in the IR-POI group and was only observed in cases with mtDNA deletions (Table 1, Table 3). The prevalence of heat and cold intolerance was similar across all subgroups (Table 3), although in the IR-POI group, these symptoms were more frequent among individuals with mtDNA deletions (Figure 1B). Furthermore, a significant association was observed for other endocrine symptoms, which occurred far more frequently in deletion-positive patients within both the IR-PCOS and IR-POI subgroups (Figure 1B).

### 3.3. Association Between GDF-15 Levels and Hormone Levels

**Figure 2:**
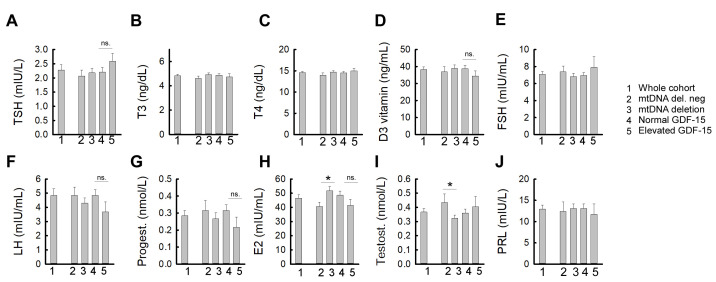
Mean levels of major hormonal parameters: (**A**) TSH, (**B**) T3, (**C**) T4, (**D**) vitamin D3, (**E**) FSH, (**F**) LH, (**G**) progesterone, (**H**) estradiol, (**I**) testosterone, (**J**) prolactin in the whole cohort (1) and subgroups without mtDNA deletions (2), with mtDNA deletions (3), in the subgroup with normal plasma GDF-15 levels (4), and with elevated GDF-15 levels (5). * = *p* < 0.05, ns = non-significant.

This study also examined the relationship between hormone levels relevant to female reproductive function and plasma GDF-15 concentrations, considering mtDNA deletion status. The mean levels of major hormonal parameters (TSH, T3, T4, vitamin D3, FSH, LH, progesterone, estradiol, testosterone, prolactin) in the entire cohort, and in the subgroups with or without mtDNA deletion, and with normal and elevated plasma GDF-15 levels are shown in Figure 2. Outliers exceeding two standard deviations from the mean were excluded. Statistical significance between the groups was assessed using analysis of variance (ANOVA). Based on the hormonal abnormalities characteristic of the chronic conditions of the patient groups studied, it was expected that AMH levels would differ significantly between all subgroups. As expected, progesterone levels showed a significant difference between the IR-PCOS and IR-POI groups, while fasting serum insulin was significantly higher in the IR-only group compared to both the IR-PCOS and IR-POI groups. No other parameters showed significant differences between the subgroups. It is worth noting that the mean age of the IR-PCOS subgroup was significantly lower than that of both the IR-only and IR-POI groups (Supplementary Table S2). To assess statistical significance, simple and multiple linear regressions were performed, alongside Spearman correlation analysis, which yields equivalent *p*-values to Pearson’s under bivariate normality. Age and BMI, known confounders of GDF-15, were included as covariates in the multivariate models. Analyses were conducted both in the total cohort and stratified by the presence or absence of mtDNA deletions to evaluate potential effects of mitochondrial dysfunction.

**Figure 3:**
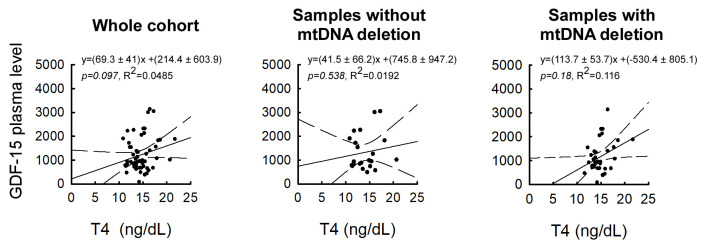
Correlation of plasma GDF-15 with T4 in patients with and without mtDNA deletions. Linear regression of plasma GDF-15 and T4 levels in the whole patient group; Linear regression of plasma GDF-15 and T4 hormone levels in the subgroup without mtDNA deletion; Linear regression of plasma GDF-15 and T4 levels in the subgroup with single or multiple mtDNA deletions. Solid lines indicate linear regression; dashed lines represent the 95% confidence interval.

Among thyroid function parameters, only T4 showed an association with plasma GDF-15 levels. However, average thyroid hormone concentrations did not differ between the investigated subgroups (Figure 3). In the total cohort, multiple linear regression identified T4 as an independent predictor of GDF-15 (β = 88.4, *p* = 0.035), while age and BMI were not significant covariates; the overall model reached statistical significance (F(3,54) = 2.91, *p* = 0.043, R^2^ = 0.139), despite violations of normality and homoscedasticity. In the deletion-negative subgroup, the model was also significant (F(3,18) = 4.60, *p* = 0.015, R^2^ = 0.434), with BMI as the only significant predictor (β = 69.3, *p* = 0.005), and T4 showing a non-significant trend (*p* = 0.090). In contrast, in the deletion-positive subgroup, univariate analysis showed no significant association between T4 and GDF-15 (*p* = 0.180) (Figure 3; Supplementary Table S3). When age and BMI were included in the multivariate model, T4 reached nominal significance (β = 114.9, *p* = 0.048); however, the overall model did not reach significance (*p* = 0.196, R^2^ = 0.134). The regression slope was more than twice as steep in the deletion-positive group (β = 103.6) compared to the deletion-negative group (β = 41.5), suggesting a stronger trend for a positive association between T4 and plasma GDF-15 levels in the presence of mitochondrial dysfunction, although these subgroup findings should be considered exploratory. Although not statistically significant, a steeper slope in deletion-positive women was evident only in the multivariate model (Figure 3). TSH and T3 showed no significant associations with GDF-15 in any subgroup (Supplementary Table S3).

When assessing the relationship between female reproductive hormone levels and GDF-15, significant differences were observed only for estradiol and testosterone. In the subgroup with mtDNA deletion, serum estradiol levels were significantly higher, while total testosterone levels were significantly reduced compared to those without the deletion (Figure 2, Supplementary Table S3). The levels of other reproductive hormones did not differ significantly between the subgroups. Additionally, neither simple nor multiple linear regression analyses, adjusted for age and BMI, revealed significant associations between GDF-15 and any of the reproductive hormones (Figure 2; Supplementary Table S3).

Among individuals without mtDNA deletions (*n* = 23), the multiple regression model including age, BMI, and vitamin D3 levels significantly predicted plasma GDF-15 concentrations (R^2^ = 0.353, *p* = 0.037). While none of the predictors reached statistical significance individually, the combined effect of these factors explained a meaningful proportion of variance. Notably, the direction of the association for vitamin D3 remained negative (β = −16.28, *p* = 0.368), suggesting that lower vitamin D3 levels may contribute to higher GDF-15 concentrations in this subgroup (Table 4, Supplementary Table S3).

**Figure 4:**
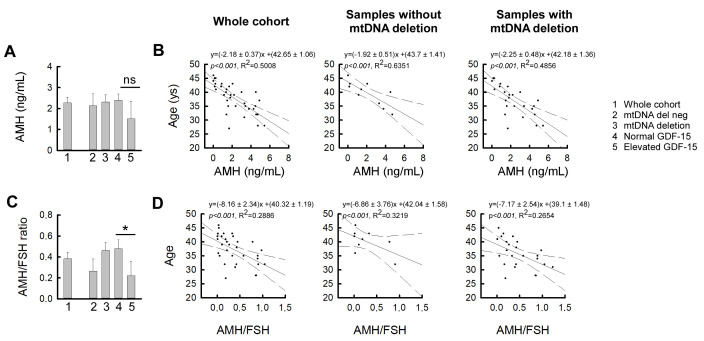
Associations between ovarian reserve markers and age in relation to mtDNA deletion and GDF-15 status. (**A**) Serum anti-Müllerian hormone (AMH) concentrations and (**C**) AMH/FSH ratios in the indicated subgroups: (1) whole cohort, (2) mtDNA deletion-negative, (3) mtDNA deletion-positive, (4) normal GDF-15, and (5) elevated GDF-15. Data are shown as mean ± SEM. Statistical analyses were performed using one-way ANOVA followed by post hoc testing; *p* < 0.05, * = *p *< 0.05, ns = not significant. (**B**,**D**) Correlation analyses between age and AMH (**B**) or AMH/FSH ratio (**D**) in the whole cohort (**left**), samples without mtDNA deletion (**middle**), and samples with mtDNA deletion (**right**). Linear regression lines with 95% confidence intervals (Solid lines indicate linear regression; dashed lines represent the 95% confidence interval) are shown. The corresponding regression equations, coefficients of determination (R^2^), and *p*-values are indicated in each panel.

Subsequently, ovarian reserve markers were analyzed to assess their relationship with mtDNA deletion status and plasma GDF-15 concentrations (Figure 4). AMH is widely used to assess ovarian reserve and is often complemented by the AMH/FSH ratio (Figure 4). To investigate their relationship with mitochondrial stress, we correlated both AMH and AMH/FSH ratio values with plasma GDF-15 levels. These analyses were restricted to the IR-only subgroup and stratified by mtDNA deletion status; the IR-PCOS and IR-POI subgroups were excluded due to their distinct hormonal profiles and pathophysiology. Although mean AMH levels tended to be lower in patients with elevated GDF-15, this difference did not reach statistical significance (Figure 4A). In contrast, within the mtDNA deletion-positive group, a significantly reduced AMH/FSH ratio was observed in individuals with elevated GDF-15 levels compared to those with GDF-15 within the normal range (*p* < 0.05; Figure 4C), suggesting a link between mitochondrial dysfunction and diminished ovarian reserve. In the investigated patient group (*n* = 35), AMH levels showed a strong inverse association with age (R^2^ = 0.501, *p* < 0.001; β = −2.18), confirming its utility as a marker of reproductive aging. This association remained significant when stratified by mtDNA deletion status: both deletion-negative (R^2^ = 0.635, *p* = 0.006; β = −1.93) and deletion-positive (R^2^ = 0.486, *p* < 0.001; β = −2.25) subgroups demonstrated strong inverse correlations, with a steeper slope in the deletion-positive group, indicating a more rapid age-related decline in AMH.

Similarly, the AMH/FSH ratio was inversely associated with age in the investigated patient group (*n* = 32; R^2^ = 0.289, *p* = 0.002; β = −8.16), and this relationship remained significant in the deletion-positive subgroup (R^2^ = 0.265, *p* = 0.010; β = −7.17). A comparable trend was observed in the deletion-negative group (β = −6.86), although it did not reach significance (R^2^ = 0.322, *p* = 0.111; power = 0.351). Notably, both the slope and intercept were lower in the deletion-positive group, suggesting that comparable AMH/FSH values occurred at younger ages and declined more steeply over time, consistent with a pattern of accelerated reproductive aging in the presence of mitochondrial dysfunction.

Among individuals without mtDNA deletions (*n* = 23), the multiple regression model including age, BMI, and vitamin D3 levels significantly predicted plasma GDF-15 concentrations (R^2^ = 0.353, *p* = 0.037). While none of the predictors reached statistical significance individually, the combined effect of these factors explained a meaningful proportion of variance. Notably, the direction of the association for vitamin D3 remained negative (β = −16.28, *p* = 0.368), suggesting that lower vitamin D3 levels may contribute to higher GDF-15 concentrations in this subgroup (Table 4; Supplementary Table S3).

## 4. Discussion

This study investigates the frequency and distribution of various organ system symptoms and their association with mitochondrial dysfunction in female patients with reproductive endocrine disorders. In addition, the relationship between relevant serum hormone levels affecting female reproductive function and plasma GDF-15 levels, as well as their effect on the presence or absence of mtDNA deletions, was also analyzed.

Among our investigated patients, the prevalence of multi-organ involvement was higher in individuals with mtDNA deletions than in those without. Notably, our previous analysis revealed that the overall frequency of mtDNA deletions in our cohort was significantly higher than in age-matched healthy controls (60.5% vs. 9.7%; χ^2^ = 29.1, *p* < 0.05) [3]. The present findings independently validate and extend our previously published results [3], confirming that the elevated frequency of mtDNA deletions and increased plasma GDF-15 levels observed in insulin-resistant women represent reproducible markers of mitochondrial dysfunction and reproductive aging. This finding lends support to the hypothesis that mitochondrial genomic instability could contribute to the development of insulin resistance. This finding is consistent with the pathophysiology of primary mitochondrial diseases, which frequently present with multisystemic involvement of varying severity [27]. When interpreting our findings, we regarded mtDNA deletions as structural alterations and ‘mitochondrial dysfunction’ as functional impairment. Consistent with the literature, multiple deletions are widely considered surrogate markers of mitochondrial dysfunction as they have repeatedly been linked to reduced respiratory chain activity and impaired OXPHOS capacity [28]. In our cohort, patients with mtDNA deletions exhibited a higher frequency of muscular, gastrointestinal, neuropsychiatric, autoimmune, and other endocrine symptoms. However, a statistically significant increase was observed only in the category of other endocrine disorders (χ^2^ = 5.02, *p* < 0.05) (Figure 1B). These results imply that mitochondrial dysfunction could contribute to the complexity and phenotypic diversity of clinical manifestations in insulin-resistant patients. A significantly higher frequency of involvement of more than five organ systems was also observed in individuals with mtDNA deletions. This finding further supports the link between structural mitochondrial damage and systemic symptom burden (Supplementary Table S1).

The multisystemic nature of insulin resistance (IR), polycystic ovary syndrome (PCOS), and premature ovarian insufficiency (POI) has recently garnered increasing attention. In PCOS, hyperandrogenism is closely linked to metabolic dysfunction, primarily insulin resistance, which affects 60–80% of patients and may drive broader systemic complications [29]. IR itself is associated with obesity, dyslipidemia, hypertension, and increased cardiovascular risk [30], while elevated inflammatory markers such as CRP and IL-6 reflect a chronic low-grade inflammatory state that may further amplify multiorgan involvement [31].

In this context, our subgroup analysis revealed that deletion-positive patients in the IR-PCOS group more frequently exhibited gastrointestinal, neurological, and exercise intolerance symptoms than patients in other subgroups (see Figure 1A,B). Although formal comparisons were limited by the small sample size, a statistically significant association with mtDNA deletions was observed for psychiatric symptoms only (Figure 1B). This suggests a potential interaction between mitochondrial dysfunction and neuropsychiatric vulnerability in this phenotype. These observations are consistent with emerging evidence implicating mitochondrial dysfunction, particularly multiple mtDNA deletions, in the pathogenesis of PCOS. Here, it may exacerbate insulin resistance, promote hyperandrogenism, and contribute to systemic inflammation [29,32]. While the cross-sectional design of our study precludes causal inference, the clustering of symptoms in IR-PCOS patients with mtDNA deletions indicates that mitochondrial dysfunction could be a significant factor contributing to disease heterogeneity.

Using age-specific cut-off values from a recent meta-analysis [26], 12 patients were identified with elevated plasma GDF-15 levels, most of whom also had mtDNA deletions and/or elevated BMI. Although numbers were insufficient for statistical analysis, this overlap suggests that mitochondrial dysfunction may contribute to systemic stress signaling. Thermoregulatory symptoms and autoimmune disorders were more prevalent in this subgroup, though these differences were not statistically significant. However, the immunomodulatory effects of GDF-15, such as its impact on lymphocyte proliferation, cytokine suppression, and macrophage activation, may explain the observed trend of higher autoimmune prevalence in patients with elevated GDF-15 levels [33,34,35,36]. These mechanisms may underlie the association we observed between elevated GDF-15 and increased autoimmune burden. Taken together, our findings support the view that mtDNA deletions and elevated GDF-15 act as complementary markers of multisystem involvement in insulin-resistant conditions, reflecting the complex endocrine, immune, and neurovegetative phenotypes of IR, PCOS, and POI.

Next, we investigated the relationship between plasma GDF-15 levels, reproductively relevant hormones, and mtDNA deletions. In the total cohort, T4 emerged as a significant independent predictor of GDF-15, even after adjusting for age and BMI. This robust association suggests that thyroid hormones may influence mitochondrial stress signaling in insulin-resistant women. In subgroup analyses, BMI was the strongest predictor in deletion-negative patients, whereas in deletion-positive patients, the regression slope for T4 was more than twice as steep, indicating a possible stronger association. However, since the overall model did not reach significance in this subgroup, these findings should be regarded as exploratory. The observation of a T4–GDF-15 link is biologically plausible, given that thyroid hormones are well-established regulators of mitochondrial biogenesis and oxidative phosphorylation. Both experimental and clinical studies of hyperthyroidism have demonstrated that increased T4 levels lead to higher GDF-15 expression [37,38,39,40,41]. This association has not previously been reported in women with insulin resistance. Therefore, subtle variations in thyroid status may amplify mitochondrial stress signaling and contribute to systemic manifestations, particularly in endocrine-metabolic conditions such as insulin resistance (IR), polycystic ovary syndrome (PCOS), and primary ovarian insufficiency (POI), where thyroid dysfunction and mitochondrial impairment may coexist.

Vitamin D3 did not show consistent associations with GDF-15 across the cohort. Stratified analyses suggested opposite trends in deletion-negative versus deletion-positive patients, which may have canceled each other out in the pooled data. Given the established role of vitamin D in maintaining mitochondrial function and reducing oxidative stress [42,43], these patterns may reflect context-dependent interactions with stress pathways. A positive trend in deletion-positive patients could indicate a compensatory mechanism, whereas vitamin D may be insufficient to counteract GDF-15 upregulation in metabolically burdened states. Taken together with the stronger T4–GDF-15 correlation in deletion-positive patients, our findings suggest that mitochondrial dysfunction amplifies the endocrine–metabolic effects of thyroid hormones and modulates vitamin D-related stress responses. To our knowledge, this is the first study to link subtle changes in the thyroid gland and vitamin D dynamics with mitochondrial stress signaling in insulin-resistant women.

Given the established links between estradiol, mitochondrial activity [44,45,46], and GDF-15 as a biomarker of cardiovascular risk [47,48,49,50], our findings suggest that mitochondrial dysfunction may contribute to long-term cardiometabolic vulnerability in insulin-resistant women. Notably, patients with mtDNA deletions showed lower testosterone levels, consistent with the role of mitochondrial integrity in steroidogenesis and oxidative balance [51]. Testosterone deficiency may further weaken mitochondrial resilience, creating a vicious cycle that exacerbates both metabolic and reproductive disturbances and promotes features of accelerated aging [52,53,54,55]. In addition, women with mtDNA deletions and elevated GDF-15 displayed lower AMH/FSH ratios, indicating impaired ovarian reserve. This aligns with prior evidence linking mitochondrial abnormalities—including reduced mtDNA copy number, mtDNA mutations, and OXPHOS defects—to diminished ovarian reserve and reproductive potential [56,57]. To minimize bias from PCOS and POI, where AMH levels are altered in opposite directions, these analyses were restricted to the IR-only subgroup. While overt aging features were not yet evident in our relatively young IR-POI subgroup, the high prevalence of mtDNA deletions supports the hypothesis that mitochondrial dysfunction may be an early driver of POI pathogenesis [54,58,59].

In our findings, evidence of accelerated reproductive aging was most prominent in the subgroup with mitochondrial DNA deletions, as reflected by significantly lower AMH/FSH ratios and steeper age-related declines in both AMH and AMH/FSH values. These results support and extend previous work by our group, indicating that elevated GDF-15 levels and the presence of mtDNA deletions may reflect underlying mitochondrial stress and metabolic dysregulation, potentially serving as early markers of accelerated aging in insulin-resistant women [3]. Importantly, our observation concerns not the well-established physiological roles of AMH and FSH, but their integrative evaluation with mitochondrial biomarkers. This highlights a pattern of accelerated reproductive aging in women with mtDNA deletions.

## 5. Conclusions

In summary, in insulin-resistant women—including those with PCOS and POI—mtDNA deletions and elevated GDF-15 levels track with multi-organ involvement and with hormonal alterations relevant to reproductive function. In the total cohort, free thyroxine (T4) was independently associated with GDF-15, supporting a link between thyroid function and mitochondrial stress. In women with mtDNA deletions, a steeper but non-significant trend was observed, suggesting that mitochondrial damage may further amplify thyroid-driven stress signaling, although this requires confirmation.

Deletion carriers also exhibited lower AMH/FSH ratios and steeper age-related declines in ovarian reserve markers, consistent with features of accelerated reproductive aging. Taken together, these findings support the utility of mtDNA deletions and GDF-15 as complementary indicators of mitonuclear stress with potential relevance to systemic and reproductive health. Given the retrospective design, modest subgroup sizes, and the absence of a priori power calculation, our results should be viewed as hypothesis-generating; prospective, adequately powered studies are warranted to validate these associations and to clarify causality.

## 6. Strengths and Limitations

This study is one of the first to clarify the link between mitochondrial dysfunction and reproductive hormone levels and symptoms affecting multiple organ systems.

The study emphasizes that mitochondrial dysfunction is a significant factor in the development of reproductive disorders, highlighting its potential role in conditions like insulin resistance, polycystic ovary syndrome (PCOS), and premature ovarian insufficiency (POI).

The study reveals complex hormonal interactions, particularly in subgroups of patients. This indicates that mitochondrial dysfunction may affect hormonal profiles differently depending on the underlying condition.

The involvement of multiple organs in patients with mtDNA deletion suggests a systemic impact of mitochondrial dysfunction, indicating that it may affect not only reproductive health but also general health.

A limitation of the present work is the relatively small sample size of the IR-PCOS and IR-POI subgroups, which reduces statistical power. Therefore, subgroup analyses should be regarded as exploratory and hypothesis-generating, while the main associations (e.g., between mtDNA deletions, GDF-15, and reproductive aging markers) were also evident in the total cohort. In addition, the absence of a priori sample size estimation means that type II errors cannot be fully excluded.

However, adjustments were made for age and BMI; other potential confounders, including medication use, inflammatory status, lifestyle factors, comorbidities, and environmental exposures, were not systematically controlled. Together with cohort heterogeneity (IR, PCOS, and POI), this may limit condition-specific interpretations. Furthermore, as the study population is Hungarian and North-Eastern European Caucasian, the generalizability of the findings is geographically and ethnically restricted.

Despite these limitations, the main associations between mtDNA deletions, GDF-15, and reproductive aging markers remained consistent across the total cohort, supporting their hypothesis-generating significance.

## Figures and Tables

**Table 1:** Organ system–specific complaints and symptoms in patient subgroups stratified by mtDNA deletion status and GDF-15 plasma levels. Percentage of specific organ system complaints and symptoms in whole patient cohort (*n* = 81) (1), in the subgroup without mtDNA deletion (*n* = 31) (2), in the subgroup with single or multiple mtDNA deletions (*n* = 50) (3), in the subgroup with normal GDF-15 plasma levels (expressed as pg/mL) (*n* = 69) (4) and in the subgroup with elevated GDF-15 plasma levels (*n* = 12), based on self-completion questionnaire responses and available medical record data. The significance between pairs of groups was calculated using the chi-square and Fisher’s exact tests. Significant differences are marked in bold (*p* < 0.05). (Abbreviations: mtDNA, mitochondrial DNA; del., deletion; NA, not applicable; GI, gastrointestinal; TIA, transient ischemic attack.).

Clinical Symptoms	Whole Cohort	mtDNA del. Neg	mtDNA del. Pos	Fisher Exact Test	Chi^2^	Normal GDF-15	Elevated GDF-15	Fisher Exact Test	Chi^2^
Exercise intolerance	40.0% (32/81)	29.4% (9/31)	42.1% (21/50)	0.34	1.38	39.7% (27/69)	41.70% (5/12)	1	0.03
Visual impairment	8.2% (7/81)	8.8% (3/31)	7.0% (4/50)	0.71	0.08	8.2% (6/69)	8.30% (1/12)	1	0.01
Hearing impairment	3.5% (3/81)	0.0% (0/31)	5.3% (3/50)	0.28	NA	4.1% (3/69)	0.00% (0/12)	1	NA
GI symptoms	43.5% (35/81)	32.4% (10/31)	45.6% (23/50)	0.25	1.49	43.8% (30/69)	41.70% (5/12)	1	0.01
Cardiac involvement	14.1% (11/81)	17.6% (5/31)	10.5% (5/50)	0.49	0.66	16.4% (11/69)	0.00% (0/12)	0.21	NA
TIA/Stroke	0.0% (0/81)	0.0% (0/31)	0.0% (0/50)	1	NA	0.0% (0/69)	0.00% (0/12)	1	NA
Psychomotor delay	9.4% (8/81)	2.9% (1/31)	12.3% (6/50)	0.24	1.86	9.6% (7/69)	8.30% (1/12)	1	0.04
Neurological symptoms	7.1% (6/81)	5.9% (2/31)	7.0% (4/50)	1	0.05	8.2% (6/69)	0.00% (0/12)	0.59	NA
Psychiatric involvement	35.3% (29/81)	26.5% (8/31)	36.8% (18/50)	0.46	0.91	37.0% (26/69)	25.00% (3/12)	0.53	0.72
Autoimmune involvement	25.9% (21/81)	17.6% (5/31)	28.1% (14/50)	0.29	1.50	24.7% (17/69)	33.30% (4/12)	0.5	0.40
Heat or cold intolerance	56.5% (46/81)	50.0% (16/31)	54.4% (27/50)	1	0.12	56.2% (39/69)	66.6% (8/12)	0.75	0.45
Other endocrine symptoms	45.7% (38/81)	29% (9/31)	57.1% (29/50)	**0.013**	**6.45**	46.4% (32/69)	41.7% (5/12)	1	0.09

**Table 2:** Concurrent organ system symptoms in patients with elevated GDF-15 levels. Frequency of concurrent symptoms in different organ systems in patients with elevated GDF-15 levels based on self-completion questionnaire responses and available medical record data (Abbreviations: P1–12, Patient 1–12).

Clinical Symptoms	P1	P2	P3	P4	P5	P6	P7	P8	P9	P10	P11	P12
Exercise intolerance (*n* = 5)	+	−	+	+	−	−	−	−	−	+	+	−
Visual impairment (*n* = 1)	−	+	−	−	−	−	−	−	−	−	−	−
Hearing impairment (*n* = 0)	−	−	−	−	−	−	−	−	−	−	−	−
GI symptoms (*n* = 5)	+	−	−	+	+	+	+	−	−	−	−	−
Cardiac involvement (*n* = 1)	−	−	−	−	−	−	−	+	−	−	−	−
TIA/Stroke (*n* = 0)	−	−	−	−	−	−	−	−	−	−	−	−
Psychomotor delay (*n* = 1)	+	−	−	−	−	−	−	−	−	−	−	−
Neurological symptoms (*n* = 0)	−	−	−	−	−	−	−	−	−	−	−	−
Psychiatric involvement (*n* = 3)	+	−	−	+	+	−	−	−	−	−	−	−
Autoimmune involvement (*n* = 4)	+	−	−	+	−	+	+	−	−	−	−	−
Heat or cold intolerance (*n* = 8)	+	+	+	+	+	+	−	−	−	+	−	+
Other endocrine symptoms (*n* = 5)	−	−	−	+	+	+	+	−	−	−	+	−

**Table 3:** Organ system–specific complaints in patient subgroups with IR, IR+PCOS, and IR+POI. Percentage of specific organ system complaints and symptoms across subgroups. Chi-square and Fischer exact test used for significance. In statistical analyses, both the IR-PCOS and IR-POI were compared to the IR-only. NA, not applicable.

Clinical Symptoms	IR	IR-PCOS	Fisher Exact Test	Chi^2^	IR-POI	Fisher Exact Test	Chi^2^
		IR vs. IR-PCOS			IR vs. IR-POI		
Exercise intolerance	40.8% (20/49)	42.1% (8/19)	1	0.01	30.8% (4/13)	0.44	0.75
Visual impairment	8.2% (4/49)	10.5% (2/19)	0.67	0.10	0.0% (0/13)	NA	0.57
Hearing impairment	4.1% (2/49)	0.0% (0/19)	1	NA	7.7% (1/13)	0.29	0.51
GI symptoms	36.7% (18/49)	52.6% (10/19)	0.28	1.43	38.5% (5/13)	0.01	1
Cardiac involvement	10.2% (5/49)	26.3% (5/19)	0.13	2.83	0.0% (0/13)	NA	0.57
TIA/Stroke	0.0% (0/49)	0.0% (0/19)	1	NA	0.0% (0/13)	NA	1
Psychomotor delay	10.2% (5/49)	15.8% (3/19)	0.68	0.41	0.0% (0/13)	NA	0.57
Neurological symptoms	6.1% (3/49)	10.5% (2/19)	0.61	1.03	0.0% (0/13)	NA	1
Psychiatric involvement	38.8% (19/49)	26.3% (5/19)	0.41	0.93	38.5% (5/13)	0.00	1
Autoimmune involvement	26.5% (13/49)	26.3% (5/19)	1	0.01	15.4% (2/13)	0.70	0.49
Heat or cold intolerance	57.1% (28/49)	52.6% (10/19)	0.79	0.11	53.8% (7/13)	0.05	1
Other endocrine symptoms	46.9% (23/49)	36.8% (7/19)	0.23	0.81	36.8% (7/13)	0.66	0.79

**Table 4:** Results of multiple linear regression analysis for T4, vitamin D3, and total testosterone. Values are unstandardized coefficients (b) with standard errors (SE), t-statistics (t), and two-tailed *p*-values for each predictor. “Model p” denotes the omnibus F-test *p*-value; “Model R^2^” is the coefficient of determination. Subgroups: **Del-neg:** mtDNA deletion-negative; **Del-pos**: mtDNA deletion-positive. *N* indicates the number of cases with complete data for the given model. Statistical significance was set at *p* < 0.05.

Predictor	Subgroup	b (Coeff.)	SE	t	*p*-Value	Model p	Model R2
T4	Total (*n* = 58)	88.39	40.832	2.165	0.035	0.043	0.139
T4	mtDNA del. neg. (*n* = 22)	99.565	55.612	1.79	0.09	0.015	0.434
T4	mtDNA del. pos. (*n* = 36)	114.886	55.877	2.056	0.048	0.196	0.056
Vitamin D3	mtDNA del. neg. (*n* = 22)	−10.187	10.488	−0.971	0.344	0.035	0.402
Testosterone	mtDNA del. pos. (*n* = 20)	3567.303	1281.813	2.783	0.013	0.016	0.096

## Data Availability

The original contributions presented in this study are included in the article/Supplementary Materials. Further inquiries can be directed to the corresponding author.

## Supplementary Materials

The following supporting information can be downloaded at: https://www.mdpi.com/article/10.3390/life15111744/s1.

## Abbreviations

The following abbreviations are used in this manuscript:

Acyl-CoA	Acyl Coenzyme A
ANOVA	Analysis of variance
AMH	Anti-Müllerian Hormone
ATP	Adenosine Triphosphate
BMI	Body Mass Index
CI	Confidence Interval
DAG	Diacylglycerol
DNA	Deoxyribonucleic Acid
ELISA	Enzyme-Linked Immunosorbent Assay
FSH	Follicle-Stimulating Hormone
GDF-15	Growth Differentiation Factor 15
GI	Gastrointestinal
HOMA	Homeostatic Model Assessment
IR	Insulin Resistance
LH	Luteinizing Hormone
mtDNA	Mitochondrial DNA
PBS	Phosphate-Buffered Saline
PCOS	Polycystic Ovary Syndrome
POI	Premature Ovarian Insufficiency
ROS	Reactive Oxygen Species
S.E.M.	Standard Error of the Mean
T2DM	Type 2 diabetes mellitus
T3	Triiodothyronine
T4	Thyroxine
TGF-β	Transforming Growth Factor Beta
TIA	Transient Ischemic Attack
TSH	Thyroid-Stimulating Hormone
UV	Ultraviolet
