# Supplementary material for: Mitochondrial DNA Deletions and Plasma GDF-15 Protein Levels Are Linked to Hormonal Dysregulation and Multi-Organ Involvement in Female Reproductive Endocrine Disorders
Source: Life (Basel). 2025 Nov 13;15(11):1744. doi: 10.3390/life15111744 (PMC12653276; doi:10.3390/life15111744)
Supplement: Supplementary file 1 [file life-15-01744-s001.zip › Supplementary Document 1 (Mitochondrial involvement questionnaire_ENG).pdf]

Mitochondrial involvement – clinical questionnaire

The Department of Obstetrics and Gynecology at Semmelweis University and the Institute of Genomic Medicine and Rare Diseases at Semmelweis University are launching a new research project focused on comprehensive clinical and genetic examination of patients with symptoms indicating multiorgan involvement, who have insulin resistance, polycystic ovary syndrome (PCOS), or premature ovarian failure (POI).
By answering the following screening questions, we will get an overview of whether you may have a mitochondrial disease. If, based on your answers to the questions, we believe that you may be eligible for selection, we will contact you shortly at one of the contact details provided.
Participation in the study may require additional doctor-patient visits.

Name:

Date of birth:

Social security number:

Phone number:

Email address:

Height: Weight:

Regularly taken medications:

You visited our clinic because of the following problem:

Do you currently have, or have you had in previous years, any complaints related to muscle function (muscle weakness, muscle soreness after physical exertion, more pronounced than average fatigue after physical exertion, regular muscle cramps)?

Do you have any known vision or hearing impairments that require regular care from an ophthalmologist or ENT specialist?

Do you have any symptoms indicating digestive problems (diarrhea, food intolerance, bloating, loss of appetite, weight loss, childhood weight deficiency, short stature, etc.)?

Do you have heart disease (known cardiac arrhythmia, cardiomyopathy)?

Have you ever had a TIA episode/stroke (transient ischemic attack or cerebral infarction)?

During your early childhood, did specialists diagnose you with delayed motor or speech development?

Do you have any other neurological symptoms (movement coordination disorder - ataxia, peripheral neuropathy)?

Have you ever had psychiatric symptoms (memory impairment, persistent mood disorders, depression, psychotic episodes)?

Do you have any known autoimmune diseases?

Do you have any complaints related to heat or cold intolerance (difficulty tolerating hot or cold temperatures)?

Do you have any other endocrinological symptoms that we have not asked about yet?

Has anyone in your family ever had any of the following symptoms:

- muscle weakness
- intolerance to physical exertion
- vision impairment, vision loss
- hearing impairment, hearing loss
- heart disease
- cerebral infarction
- epileptic seizures
- neurodegenerative diseases (e.g. Parkinson's disease, Alzheimer's disease, other forms of dementia)
- memory impairment
- psychiatric disorders (depression, psychosis, autistic symptoms)
- ataxia (movement coordination disorder originating in the cerebellum)
- peripheral neuropathy (nerve damage along the peripheral nerves)
- short stature
- early childhood (psychomotor) developmental delay
- floppy baby syndrome (decreased muscle tone after birth or in early childhood)
- insulin resistance
- diabetes
- thyroid disease
- other endocrine disorders
- lipomatosis (presence of numerous subcutaneous fat deposits)
- other important conditions (e.g., cancer)
