# Supplementary material for: Mitochondrial DNA Deletions and Plasma GDF-15 Protein Levels Are Linked to Hormonal Dysregulation and Multi-Organ Involvement in Female Reproductive Endocrine Disorders
Source: Life (Basel). 2025 Nov 13;15(11):1744. doi: 10.3390/life15111744 (PMC12653276; doi:10.3390/life15111744)
Supplement: Supplementary file 1 [file life-15-01744-s001.zip › Supplementary Document 2 (Mitochondrial involvement questionnaire_HUN).pdf]

Mitokondriális érintettség – klinikai kérdőív

A Semmelweis Egyetem Szülészeti és Nőgyógyászati Klinika és a Semmelweis Egyetem Genomikai Medicina és Ritka Betegségek Intézete együttműködésében indul egy új kutatás, melynek lényege a többszervi érintettségre utaló tünetekkel rendelkező betegek kórfolyamatának átfogó klinikai és genetikai vizsgálata, akinél az inzulinrezisztencia, policisztás ovárium szindróma (PCOS), vagy korai petefészek-kimerülés (POI) közül bármelyik fennáll.
Az alábbi előszűrő kérdések megválaszolásával egy áttekintést kapunk, hogy Önnél felmerül-e esetlegesen a mitokondriális betegség lehetősége. Amennyiben a kérdésekre adott válaszok alapján úgy gondoljuk, hogy Ön a beválasztásra esélyes lehet, a megadott elérhetőségek egyikén hamarosan fel fogjuk venni Önnel a kapcsolatot.
A kutatásban való részvétel plusz orvos-beteg találkozást tehet szükségessé.

Név:

Születési idő:

TAJ szám:

Telefonszám:

Email cím:

Testmagasság: Testsúly:

Rendszeresen szedett gyógyszerek:

Ambulanciánkat a következő probléma miatt kereste fel:

Jelenleg van-e Önnek, vagy az előző években fordult-e elő Önnél izomműködéssel kapcsolatos panasz (izomgyengeség, izomlázszerű érzés fizikai terhelésre, az átlagosnál kifejezettebb mértékű fáradtság fizikai terhelésre, rendszeres izomgörcsök)?

Áll-e fenn Önnél ismert látás- vagy halláskárosodás, mely miatt esetleg rendszeres gondozásban részesül szemészeten vagy fül-orr-gégészeten?

Vannak-e emésztőszervrendszeri problémára utaló tünetei (hasmenés, ételintolerancia, haspuffadás, étvágytalanság, fogyás, gyermekkori indulású testsúlybeli elmaradás, alacsonynövés stb.)?

Áll-e fenn Önnél szívbetegség (ismert szívritmuszavar, cardiomyopathia)?

Fordult-e már elő valaha Önnél TIA epizód/stroke (átmeneti agyi érelzáródással járó epizód vagy agyi infarktus)?

A kora gyermekkori éveket tekintve véleményezték-e Önnél a szakemberek mozgás- vagy beszédfejlődésbeli elmaradást?

Egyéb idegrendszeri tünet áll-e fenn Önnél (mozgáskoordinációs zavar - ataxia, perifériás neuropathia)?

Fordult-e már elő Önnél korábban pszichiátriai jellegű tünetek (memóriazavar, tartósabb hangulatzavar, depresszió, pszichotikus epizód)?

Ismert autoimmun betegsége van-e?

Vannak-e Önnek meleg vagy hideg intoleranciával kapcsolatos panaszai (nehezen viseli a meleg vagy hideg hőmérsékletet)?

Egyéb endokrinológiai tünet, amire eddig nem kérdeztünk rá, áll-e fenn Önnél?

Fordult-e elő a családjában valakinél bármelyik az alábbi tünetek közül:
- izomgyengeség
- fizikai terhelési intolerancia
- látáskárosodás, látásvesztés
- halláskárosodás, hallásvesztés
- szívbetegség
- agyi infarktus
- epilepsziás roham
- idegrendszeri leépüléssel járó (neurodegeneratív) kórképek (pl. Parkinson-kór, Alzheimer-kór, egyéb demenciák)
- memóriazavar
- pszichiátriai betegség (depresszió, pszichózis, autisztikus tünetek)
- ataxia (kisagyi eredetű mozgáskoordinációs zavar)
- perifériás neuropathia (idegkárosodás a perifériás idegek mentén)
- alacsonynövés
- kora gyermekkori (pszichomotoros) fejlődésbeli elmaradás
- ’floppy baby syndrome’ (születés utáni vagy kora gyermekkori csökkent izomtónus)
- inzulinrezisztencia
- cukorbetegség
- pajzsmirigybetegség
- egyéb endokrinológiai betegség
- lipomatosis (bőr alatti zsírcsomók nagyszámú jelenléte)
- egyéb fontos (pl. daganatos betegség)
