# Supplementary material for: Mitochondrial DNA Deletions and Plasma GDF-15 Protein Levels Are Linked to Hormonal Dysregulation and Multi-Organ Involvement in Female Reproductive Endocrine Disorders
Source: Life (Basel). 2025 Nov 13;15(11):1744. doi: 10.3390/life15111744 (PMC12653276; doi:10.3390/life15111744)
Supplement: Supplementary file 1 [file life-15-01744-s001.zip › Supplementary Table S1.pdf]

Number of affected organ systems	Whole cohort	mtDNA del. Neg	mtDNA del. Pos.	Chi2	Fisher's Exact test	Normal GDF-15	Elevated GDF-15	Chi2	Fisher's Exact test
0-5	82.7% (67/81)	96.8% (30/31)	74% (37/50)	***6.94***	***0.01***	82.6% (57/69)	83.3% (10/12)	0.0038	1
>5	17.3% (14/81)	3.2% (1/31)	26% (13/50)			17.4% (12/69)	16.7% (2/12)		

Supplementary Table S1: Distribution of patients by the number of affected organ systems according to mtDNA deletion status and plasma GDF-15 levels.

The table presents the proportion of patients with ≤5 or >5 affected organ systems in the whole cohort and stratified by mitochondrial DNA (mtDNA) deletion status (negative vs. positive) and by plasma GDF-15 concentration (normal vs. elevated). Data are shown as percentages with case numbers (n/N). A significant association was found between mtDNA deletion status and multisystemic involvement ($\chi^2 = 6.94$, $p = 0.01$), while no significant difference was observed between normal and elevated GDF-15 subgroups ($p = 1.0$).

(Abbreviations: mtDNA, mitochondrial DNA; del., deletion, Neg. – negative. Pos. – positive)
