# Supplementary material for: Mitochondrial DNA Deletions and Plasma GDF-15 Protein Levels Are Linked to Hormonal Dysregulation and Multi-Organ Involvement in Female Reproductive Endocrine Disorders
Source: Life (Basel). 2025 Nov 13;15(11):1744. doi: 10.3390/life15111744 (PMC12653276; doi:10.3390/life15111744)
Supplement: Supplementary file 1 [file life-15-01744-s001.zip › Supplementary Table S2.pdf]

	IR only	IR-PCOS	IR-POI
Age at examination	37±0.8	30.6±1.2	37.7±1.2
BMI (kg/m2)	28.9±0.9	25.4±1.5	28.2±1.9
AMH (ng/ml)	3±0.5	7.1±1	0.6±0.1
Glucose 0' (mmol/L)	5.3±0.2	5.1±0.2	5.1±0.2
Insulin 0' (µU/mL)	14.3±1.1	11.4±2.2	10.7±1.8
HOMA index	3.4±0.4	2.7±0.6	2.5±0.5
Vitamin D3 (ng/mL)	38.1±2.1	36.1±2.1	41.1±2.5
Glucose 0' (mmol/L)	5.3±0.2	5.1±0.2	5.1±0.2
Glucose 60' (mmol/L)	7.3±0.3	7±0.5	7±0.7
Glucose 90' (mmol/L)	5.6±0.3	4.7±0.1	4.4±0.2
Glucose 120' (mmol/L)	6±0.3	5.2±0.3	6±0.7
Insulin 0' (µU/mL)	14.3±1.1	11.4±2.2	10.7±1.8
Insulin 60' (µU/mL)	82.2±7.6	70.8±13.4	75.5±14
Insulin 90' (µU/mL)	38.1±2.6	25.3±3.2	26.5±2
Insulin 120' (µU/mL)	56.9±6	44.5±9.8	60±14.3
TSH (mlU/L)	2.2±0.2	2.3±0.4	2.8±0.2
T4 (ng/dL)	14.8±0.3	14.1±0.6	14.5±0.7
T3 (ng/dL)	4.9±0.2	4.5±0.3	5.1±0.3
FSH (mlU/mL)	8.5±0.7	6.1±0.3	7.5±0.9
LH (mlU/mL)	5.2±0.5	5.8±0.9	3.3±0.5
Prolactin (mlU/L)	13±0.8	13.9±2.4	12.3±1.7
Estradiol (mlU/mL)	70.2±10.1	87.8±23.8	69.7±10.3
Progesterone (nmol/L)	0.7±0.2	3.1±1	0.2±0.1
Total testosterone (nmol/L)	0.4±0.1	0.5±0.1	0.5±0.1
Free testosterone (nmol/L)	4.6±0.2	4.6±1.3	1.5±0.5
SHBG (nmol/L)	53.2±2.1	59.1±6.9	65.3±8.3
Plasma GDF-15 level (pg/mL)	1355.5±101.4	899±114.3	1138.8±195.8
