# Supplementary material for: Mitochondrial DNA Deletions and Plasma GDF-15 Protein Levels Are Linked to Hormonal Dysregulation and Multi-Organ Involvement in Female Reproductive Endocrine Disorders
Source: Life (Basel). 2025 Nov 13;15(11):1744. doi: 10.3390/life15111744 (PMC12653276; doi:10.3390/life15111744)
Supplement: Supplementary file 1 [file life-15-01744-s001.zip › Supplementary Tables with Legends.pdf]

Supplementary Materials:

Number of affected organ systems	Whole cohort	mtDNA del. Neg	mtDNA del. Pos.	Chi2	Fisher's Exact test	Normal GDF-15	Elevated GDF-15	Chi2	Fisher's Exact test
0-5	82.7% (67/81)	96.8% (30/31)	74% (37/50)	***6.94***	***0.01***	82.6% (57/69)	83.3% (10/12)	0.0038	1
>5	17.3% (14/81)	3.2% (1/31)	26% (13/50)			17.4% (12/69)	16.7% (2/12)		

Supplementary Table S1: Distribution of patients by the number of affected organ systems according to mtDNA deletion status and plasma GDF-15 levels.

The table presents the proportion of patients with ≤5 or >5 affected organ systems in the whole cohort and stratified by mitochondrial DNA (mtDNA) deletion status (negative vs. positive) and by plasma GDF-15 concentration (normal vs. elevated). Data are shown as percentages with case numbers (n/N). A significant association was found between mtDNA deletion status and multisystemic involvement ($\chi^2 = 6.94$, $p = 0.01$), while no significant difference was observed between normal and elevated GDF-15 subgroups ($p = 1.0$).

(Abbreviations: mtDNA, mitochondrial DNA; del., deletion, Neg. – negative. Pos. – positive)

	IR only	IR-PCOS	IR-POI
Age at examination	37±0.8	30.6±1.2	37.7±1.2
BMI (kg/m2)	28.9±0.9	25.4±1.5	28.2±1.9
AMH (ng/ml)	3±0.5	7.1±1	0.6±0.1
Glucose 0' (mmol/L)	5.3±0.2	5.1±0.2	5.1±0.2
Insulin 0' (µU/mL)	14.3±1.1	11.4±2.2	10.7±1.8
HOMA index	3.4±0.4	2.7±0.6	2.5±0.5
Vitamin D3 (ng/mL)	38.1±2.1	36.1±2.1	41.1±2.5
Glucose 0' (mmol/L)	5.3±0.2	5.1±0.2	5.1±0.2
Glucose 60' (mmol/L)	7.3±0.3	7±0.5	7±0.7
Glucose 90' (mmol/L)	5.6±0.3	4.7±0.1	4.4±0.2
Glucose 120' (mmol/L)	6±0.3	5.2±0.3	6±0.7
Insulin 0' (µU/mL)	14.3±1.1	11.4±2.2	10.7±1.8
Insulin 60' (µU/mL)	82.2±7.6	70.8±13.4	75.5±14
Insulin 90' (µU/mL)	38.1±2.6	25.3±3.2	26.5±2
Insulin 120' (µU/mL)	56.9±6	44.5±9.8	60±14.3
TSH (mIU/L)	2.2±0.2	2.3±0.4	2.8±0.2

T4 (ng/dL)	14.8±0.3	14.1±0.6	14.5±0.7
T3 (ng/dL)	4.9±0.2	4.5±0.3	5.1±0.3
FSH (mlU/mL)	8.5±0.7	6.1±0.3	7.5±0.9
LH (mlU/mL)	5.2±0.5	5.8±0.9	3.3±0.5
Prolactin (mlU/L)	13±0.8	13.9±2.4	12.3±1.7
Estradiol (mlU/mL)	70.2±10.1	87.8±23.8	69.7±10.3
Progesterone (nmol/L)	0.7±0.2	3.1±1	0.2±0.1
Total testosterone (nmol/L)	0.4±0.1	0.5±0.1	0.5±0.1
Free testosterone (nmol/L)	4.6±0.2	4.6±1.3	1.5±0.5
SHBG (nmol/L)	53.2±2.1	59.1±6.9	65.3±8.3
Plasma GDF-15 level (pg/mL)	1355.5±101.4	899±114.3	1138.8±195.8

Supplementary Table S2: The most important endocrine parameters and hormonal levels in the 3 subgroups within the whole patient cohort: the IR-only subgroup (first column); the IR-PCOS subgroup (second column); and the IR-POI subgroup (third column). (*: p<0,05 (IR only vs IR-PCOS); +: p<0,05 (IR only vs IR-POI); #: p<0,05 (IR-PCOS vs IR-POI))

	Multilinear regression							Spearman correlation	Linear regression	
	**Coefficient**	**Std. Error**	**t**	**P**	**VIF**	**Regression p**	**N**		**p**	**r2**
Constant: GDF-15	-1569.636	963.851	-1.629	0.109		0.043	58	ns	0.097	0.0485
**T4 total**	88.39	40.832	2.165	0.035	1.057					
Age at examination	23.093	17.506	1.319	0.193	1.046					
BMI	23.922	14.275	1.676	0.1	1.102					
Constant: GDF-15	-2531.6	1242.93	-2.037	0.057		0.015	22	ns	0.538	0.0192
**T4 del neg**	99.565	55.612	1.79	0.09	1.101					
Age at examination	14.085	21.779	0.647	0.526	1.115					
BMI	69.318	21.764	3.185	0.005	1.221					
Constant: GDF-15	-1233.83	1399.997	-0.881	0.385		0.196	36	ns	0.18	0.116
**T4 del pos**	114.886	55.877	2.056	0.048	1.039					
Age at examination	21.283	26.102	0.815	0.421	1.013					
BMI	-2.794	17.482	-0.16	0.874	1.046					
Constant: GDF-15	298.125	900.333	0.331	0.742		0.391	54	ns	0.978	0.0038
**T3 total**	-7.47	104.292	-0.0716	0.943	1.004					
Age at examination	9.988	20.201	0.494	0.623	1.04					
BMI	22.176	14.304	1.55	0.127	1.044					
Constant: GDF-15	-1113.723	1358.428	-0.82	0.424		0.088	21	ns	0.872	0.0012
**T3 del neg**	102.316	173.5	0.59	0.563	1.006					
Age at examination	9.534	27.758	0.343	0.735	1.072					
BMI	58.417	22.911	2.55	0.021	1.078					
Constant: GDF-15	1290.665	1209.571	1.067	0.295		1	33	ns	0.944	0.0002
**T3 del pos**	-6.895	129.929	-0.0531	0.958	1.025					
Age at examination	-1.192	29.057	-0.041	0.968	1.022					
BMI	-1.369	17.911	-0.0764	0.94	1.043					
Constant: GDF-15	-471.567	816.075	-0.578	0.566		0.139	58	ns	0.323	0.0155
**TSH total**	-0.0952	82.833	-0.00115	0.999	1.052					
Age at examination	32.238	19.409	1.661	0.103	1.079					
BMI	20.708	14.961	1.384	0.172	1.027					
Constant: GDF-15	-522.142	1131.111	-0.462	0.65		0.095	21	ns	0.349	0.04

**TSH del neg**	-25.356	160.995	-0.157	0.877	1.012					
Age at examination	6.264	27.408	0.229	0.822	1.063					
BMI	59.518	22.903	2.599	0.019	1.058					
Constant: GDF-15	-1235.901	1197.555	-1.032	0.311		0.179	33	ns	0.617	0.0065
**TSH del pos**	133.035	181.123	0.735	0.469	1.017					
Age at examination	61.571	30.06	2.048	0.05	1.02					
BMI	1.606	18.833	0.0853	0.933	1.002					
Constant: GDF-15	231.533	785.147	0.295	0.769		0.16	60	ns	0.137	0.0011
**Vitamin D3 (ng/mL) total**	29.429	15.645	1.881	0.065	1.058					
Age at examination	10.643	14.86	0.716	0.477	1.183					
BMI	-9.999	10.616	-0.942	0.35	1.229					
Constant: GDF-15	-483.537	1177.073	-0.411	0.686		0.037	22	ns	0.075	0.307
**Vitamin D3 (ng/mL) del neg**	-16.282	17.666	-0.922	0.368	1.320					
Age at examination	34.739	19.771	1.757	0.095	1.053					
BMI	38.988	23.794	1.639	0.118	1.333					
Constant: GDF-15	1173.801	1090.416	1.076	0.29		0.901	32	ns	0.07	0.0965
**Vitamin D3 (ng/mL) del pos**	-1.946	9.445	-0.206	0.838	1.035					
Age at examination	10.862	24.255	0.448	0.657	1.02					
BMI	-10.547	17.969	-0.587	0.561	1.022					
Constant: GDF-15	970.749	1699.404	0.571	0.572		0.882	35	ns	0.3464	0.0176
**AMH (ng/ml) total**	-37.721	119.778	-0.315	0.755	2.045					
Age at examination	11.66	38.744	0.301	0.765	2.03					
BMI	1.43	20.009	0.0715	0.943	1.021					
Constant: GDF-15	3885.762	5843.314	0.665	0.531		0.645	10	ns	0.3965	0.0008
**AMH (ng/ml) del neg**	-95.358	307.409	-0.31	0.767	3.25					
Age at examination	-88.034	120.264	-0.732	0.492	2.902					
BMI	45.317	50.235	0.902	0.402	1.214					
Constant: GDF-15	453.622	1816.749	0.25	0.805						
**AMH (ng/ml) del pos**	-3.776	146.811	-0.0257	0.98	2.231	0.653	25	ns	0.3263	0.041
Age at examination	33.95	43.931	0.773	0.448	2.085					
BMI	-12.879	23.754	-0.542	0.593	1.148					

Constant: GDF-15	1365.332	1553.016	0.879	0.387						
**AMH/FSH total**	-432.656	544.595	-0.794	0.434	1.422	0.804	32	ns	0.4298	0.0305
Age at examination	1.208	35.714	0.0338	0.973	1.411					
BMI	6.965	21.956	0.317	0.753	1.012					
Constant: GDF-15	2875.891	4261.683	0.675	0.53		0.491	9	ns	0.6009	0.053
**AMH/FSH del neg**	478.246	1181.959	0.405	0.702	1.647					
Age at examination	-77.164	92.737	-0.832	0.443	1.482					
BMI	58.085	47.054	1.234	0.272	1.139					
Constant: GDF-15	1102.871	1636.187	0.674	0.508						
**AMH/FSH del pos**	-669.928	554.304	-1.209	0.241	1.398	0.399	24	ns	0.3707	0.125
Age at examination	17.586	39.633	0.444	0.662	1.385					
BMI	-1.213	24.258	-0.05	0.961	1.03					
Constant: GDF-15	-376.421	838.694	-0.449	0.656						
**FSH total**	-12.091	25.094	-0.482	0.632	1.117	0.213	54	ns	0.798	0.00127
Age at examination	31.23	21.155	1.476	0.146	1.108					
BMI	21.74	16.574	1.312	0.196	1.043					
Constant: GDF-15	-529.677	1028.873	-0.515	0.614						
**FSH del neg**	8.488	36.659	0.232	0.82	1.158	0.145	19	ns	0.895	0.00105
Age at examination	5.881	26.938	0.218	0.83	1.221					
BMI	56.912	24.431	2.33	0.034	1.09					
Constant: GDF-15	-326.781	1329.782	-0.246	0.808						
**FSH del pos**	-26.108	34.328	-0.761	0.453	1.07	0.513	15	ns	0.612	0.00789
Age at examination	46.923	32.808	1.43	0.163	1.044					
BMI	2.875	22.418	0.128	0.899	1.027					
Constant: GDF-15	235.935	960.468	0.246	0.807						
**LH total**	-31.245	szept.32	-0.95	0.347	1.15	0.205	55	ns	0.143	0.0399
Age at examination	16.45	21.278	0.773	0.443	1.139					
BMI	22.109	16.504	1.34	0.186	1.023					
Constant: GDF-15	-267.879	1385.803	-0.193	0.849						
**LH del neg**	-13.28	50.827	-0.261	0.797	1.399	0.144	19	ns	0.303	0.0623
Age at examination	4.835	28.107	0.172	0.866	1.33					
BMI	54.402	24.804	2.193	0.044	1.125					

Constant: GDF-15	455.002	1418.373	0.321	0.75						
**LH del pos**	-38.228	45.188	-0.846	0.404	1.077	0.635	36	ns	0.28	0.0342
Age at examination	23.37	33.035	0.707	0.484	1.077					
BMI	5.99	22.51	0.266	0.792	1					
Constant: GDF-15	264.292	1300.629	0.203	0.841						
**Progesterone total**	-59.686	73.144	-0.816	0.426	1.186	0.362	21	ns	0.17	0.0966
Age at examination	10.075	29.472	0.342	0.737	1.053					
BMI	27.525	24.784	1.111	0.282	1.172					
Constant: GDF-15	-1462.929	1467.253	-0.997	0.365						
**Progesterone del neg**	1.402	64.821	0.0216	0.984	1.231	0.119	9	ns	0.392	0.106
Age at examination	13.719	31.607	0.434	0.682	1.078					
BMI	73.155	26.245	2.787	0.039	1.176					
Constant: GDF-15	2470.965	2148.275	1.15	0.283						
**Progesterone del pos**	-275.391	218.154	-1.262	0.242	1.267	0.587	12	ns	0.275	0.118
Age at examination	12.213	51.038	0.239	0.817	1.121					
BMI	-36.952	40.017	-0.923	0.383	1.215					
Constant: GDF-15	-634.201	1034.826	-0.613	0.543						
**Estradiol total**	-0.000112	0.000345	-0.323	0.748	1.106	0.15	47	ns	0.359	0.0187
Age at examination	29.506	24.244	1.217	0.23	1.086					
BMI	32.068	18.153	1.766	0.084	1.021					
Constant: GDF-15	459.709	1597.678	0.288	0.778						
**Estradiol del neg**	-4.925	13.009	-0.379	0.711	1.053	0.156	18	ns	0.533	0.028
Age at examination	-12.599	35.995	-0.35	0.732	1.065					
BMI	56.574	23.946	2.363	0.033	1.055					
Constant: GDF-15	-1743.957	1740.607	-1.002	0.327						
**Estradiol del pos**	3.512	2.432	1.444	0.162	1.089	0.279	27	ns	0.33	0.038
Age at examination	66.062	38.605	1.711	0.101	1.101					
BMI	14.488	27.276	0.531	0.6	1.012					
Constant: GDF-15	-192.987	1531.005	-0.126	0.901						
**Total testosterone total**	460.977	844.414	0.546	0.59	1.133	0.789	30	ns	0.746	0.00382
Age at examination	26.776	33.429	0.801	0.43	1.141					
BMI	11.293	18.24	0.619	0.541	1.01					
Constant: GDF-15	-194.726	6205.284	-0.0314	0.976						
**Total testosterone del neg**	-248.887	1910.058	-0.13	0.901	3.382	0.214	10	ns	0.545	0.0477
Age at examination	-2.117	141.4	-0.015	0.989	3.551					
BMI	61.652	27.087	2.276	0.063	1.111					
Constant: GDF-15	1691.597	1456.014	1.162	0.262						
**Total testosterone del pos**	3567.303	1281.813	2.783	0.013	1.435	0.016	20	ns	0.197	0.0909
Age at examination	8.026	29.741	0.27	0.791	1.334					
BMI	-64.91	19.92	-3.259	0.005	1.252					
Constant: GDF-15	174.264	1345.31	0.13	0.898						
**Prolactin total**	-27.012	22.268	-1.213	0.233	1.094	0.384	42	ns	0.244	0.0338
Age at examination	21.676	29.763	0.728	0.471	1.048					
BMI	23.829	21.279	12.jan	0.27	1.05					
Constant: GDF-15	679.705	2732.77	0.249	0.809						
**Prolactin del neg**	30.245	40.301	0.75	0.47	1.299	0.269	14	ns	0.573	0.0273
Age at examination	-36.417	56.372	-0.646	0.533	1.211					
BMI	67.558	35.793	1.887	0.088	1.08					
Constant: GDF-15	-655.376	1544.271	-0.424	0.675						
**Prolactin del pos**	-51.698	27.974	-1.848	0.077	1.218	0.148	28	ns	0.097	0.102
Age at examination	51.114	34.871	1.466	0.156	1.015					
BMI	24.746	26.997	0.917	0.368	1.222					

Supplementary Table S3: Summary of multilinear regression, Spearman's correlation, and linear regression.
